# Multimodal connectivity based eloquence score computation and visualisation for computer-aided neurosurgical path planning

**DOI:** 10.1049/htl.2017.0073

**Published:** 2017-09-14

**Authors:** Saeed M. Bakhshmand, Roy Eagleson, Sandrine de Ribaupierre

**Affiliations:** 1 Biomedical Engineering Graduate Program, University of Western Ontario, London, ON, Canada; 2 Department of Electrical and Computer Engineering, University of Western Ontario, London, ON, Canada; 3 Department of Clinical Neurological Sciences, University of Western Ontario, London, ON, Canada

**Keywords:** neurophysiology, brain, surgery, cognition, bone, biomedical MRI, medical image processing, biological tissues, multimodal connectivity based eloquence score computation, multimodal connectivity based eloquence score visualisation, computer-aided neurosurgical path planning, noninvasive assessment, cognitive importance, neurosurgical procedures, in vivo brain imaging modalities, impact damage, skull, multimodal metrics, intervened grey matter volume, axonal fibre numbers, anatomical networks, functional networks, solution space, visually representing connectional cost, brain networks, resting state functional magnetic resonance imaging, fMRI, deterministic tractography, rehning traditional heuristics, resected tissue, neuroimaging modalities, related anatomical landmarks

## Abstract

Non-invasive assessment of cognitive importance has been a major challenge for planning of neurosurgical procedures. In the past decade, in vivo brain imaging modalities have been considered for estimating the ‘eloquence’ of brain areas. In order to estimate the impact of damage caused by an access path towards a target region inside of the skull, multi-modal metrics are introduced in this paper. Accordingly, this estimated damage is obtained by combining multi-modal metrics. In other words, this damage is an aggregate of intervened grey matter volume and axonal fibre numbers, weighted by their importance within the assigned anatomical and functional networks. To validate these metrics, an exhaustive search algorithm is implemented for characterising the solution space and visually representing connectional cost associated with a path initiated from underlying points. In this presentation, brain networks are built from resting state functional magnetic resonance imaging (fMRI) and deterministic tractography. their results demonstrate that the proposed approach is capable of refining traditional heuristics, such as choosing the minimal distance from the lesion, by supplementing connectional importance of the resected tissue. This provides complementary information to help the surgeon in avoiding important functional hubs and their anatomical linkages; which are derived from neuroimaging modalities and incorporated to the related anatomical landmarks.

## 1 Introduction

Planning an access trajectory towards central nervous system lesions or tumours often demands careful examination of the functional importance for the tissue surrounding the lesion and across the considered path. Failure to assess the impact of tissue damage along the candidate trajectory may result in severe cognitive, perceptual, motor, or language deficits [1]. In order to minimise the damage to the healthy tissue relying on preoperative imaging, one heuristic is to choose the path that minimises the length of the access path [2] or distance from critical vessels [3]. However, these consideration alone might not be enough. Depending on the position of the lesion, the surgeon might have to choose between causing a deficit in order to remove the whole lesion or leaving it untouched. Going through an eloquent area can result in a measurable functional deficit; however, currently it is difficult to predict the amount of the deficit.

This decision-making process becomes even more complicated once the high inter-individual variability of the brain networks is taken into consideration. Growing evidence from missing functional hubs in computational models [4] to experimental studies on the effects of brain injuries on postoperative neuropsychological deficits [5], suggests the importance of mapping the brain activity and network integrity around planned resections and focal lesions might prevent potential adverse outcomes of neurosurgical treatments. This need motivates the use of non-invasive brain imaging modalities to assess connectional aspects of the underlying tissue based on objective metrics derived from structural and functional brain networks.

Currently, planning a preoperative neurosurgical path is solely dependent on traditional and readily identifiable eloquent brain regions observed by neurosurgeon (e.g. insula, language or motor areas), regardless of individual differences that might arise from multimodal brain maps. Although there has been previous efforts to include multiple brain imaging modalities (e.g. fMRI and diffusion tensor imaging, DTI) into the automatic planning of trajectories [6–8] or employing functional connectivity (FC) to explore network status of the lesion areas [9], most of the studies have focused on avoiding vascular system [10, 11]. There have been some recent studies showing importance of the connectome in brain surgery [12]; however, this application merits more research studies to develop interactive techniques (such as [13]) to probe for functional organisation and predict long-term outcomes of resecting a tissue segment using non-invasive imaging modalities [14].

Integrating proposed method into the neurosurgical workflow assists neurosurgeons to modify their surgical access paths according to the individual patient networks. Our method is capable of mapping graph theoretical network measures of the whole brain to their anatomical counterparts, through construction and visualisation of conducted trajectory and computational estimation of its cognitive damage. This way, the surgeon is able to visualise a planned trajectory and assess its overall damage (also known as score) on structural and functional networks of any individual, decreasing the potential deficits.

## 2 Imaging data description

Dataset used in this study is collected with approval from research ethics board of UWO and consists of resting state (RS) fMRI, T1 anatomical and diffusion weighted imaging (DWI) scans acquired from healthy subjects in a 3T scanner. The RS-fMRI data comprised one session of 140 scans with a TR of 2100 ms and isotropic voxel size of 2.5 mm. Preprocessing steps are carried out in SPM [http://www.fil.ion.ucl.ac.uk/spm/] and include realignment, coregistration and smoothing with default parameters of the tool. FMRI volumes were parcellated according to Desikan Killiany (DK) atlas by an VASSET pipeline [https://github.com/akhanf/vasst-dev] and average of the time series from grey matter (GM) areas were extracted. For the computation of FC a MATLAB implementation of Dynamic FC [15] was considered and first eigenconnectivity matrix (matrix with largest eigenvalue) was chosen as the main FC matrix.

DWI scans have 64 gradient directions (138 bidirectional scans) and unweighted *B* = 0 scans (*b* = 0 S/mm^2^) with an isotropic voxel size of 2 mm. Distortion and motion artefacts were corrected using eddy and topup in FSL [https://fsl.fmrib.ox.ac.uk/fsl/fslwiki/]. DTI conversion and whole fibre tract generation (tractography) was carried out in 3D Slicer [https://www.slicer.org/]. Structural connectivity (SC) matrix is generated from deterministic tractography with regards to DK atlas parcellation of 70 regions over the GM. [16].

## 3 Methods

**Fig. 1:**
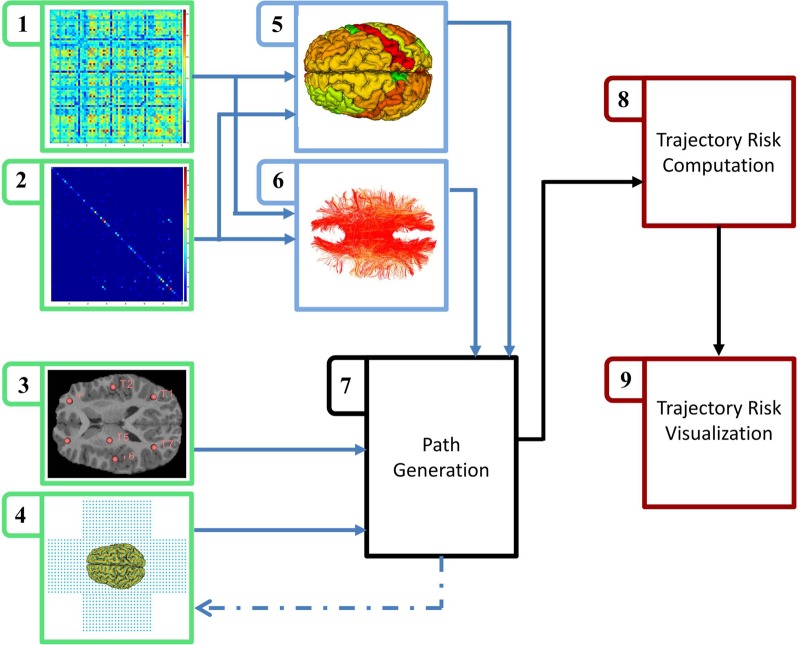
Overview of the multimodal connectivity based preoperative neurosurgical path planning

A graphical overview of the proposed workflow is given in Fig. 1. Steps (1)–(4) are the main inputs to the system. Steps (1) and (2) are FC and SC matrices, respectively. Step (3) is a collection of target points inside the central nervous system. Step (4) is a 3D box surrounding the brain, sampled with a grid of points with adequate resolution to build a search space. Steps (5) and (6) are construction of eloquence scores for the GM regions and white matter (WM) fibres. Step (7) serves to create the surgical path and isolate the GM and WM tissue impacted by the chosen path. Steps (8) and (9) compute and hold results of an iterative process that iterates between Steps (4) and (7) to cover all assigned paths from a given box in Step (4) towards a target point in Step (3).

### 3.1 Computation of eloquence scores

The objective of eloquence metric is to represent connectivity role of the underlying tissue and formulate a new approach to assess cognitive importance of the brain areas [17]. Brain networks (also known as brain connectivity) demonstrate areas of the brain that are linked either in functional or structural domains. Functional connectivity represents statistical similarities that can be extracted from brain activity recorded by functional neuroimaging methods, whereas structural connectivity shows physical linkages that exist between GM areas through WM fibres. Therefore, these two major connectivity types are mapped to the associated tissue according to the atlas that has been used to compute the connectivity matrices. In other words, the anatomical location of the GM region that represents the network node was assigned a network measure to indicate its importance in the given network. Same principle has been applied to WM fibres, only difference being that end points of the fibres are considered to find out which node of brain network they belong to. Overall, we defined four eloquent scores from measurements based on graph theoretical brain networks: structural score (SS) and functional score (FS) for GM regions and WM fibres. ${\rm FCG}{\rm M}_k$ is the functional component of an eloquence score for a GM area labelled as *k* and is defined as

(1) $${\rm FCG}{\rm M}_k = \sum\limits_{\,j = 1}^N \vert {\rm FC}\lpar k\comma \; j\rpar \vert \eqno\lpar 1\rpar $$

where FC(*k*, *j*) is the row *k* and column *j* of FC matrix. Similarly, ${\rm SCG}{\rm M}_k$ is the structural component of the eloquence score for the same GM area computed with the following equation:

(2) $${\rm SCG}{\rm M}_k = \displaystyle{{\sum\nolimits_{\,j = 1}^N {\vert {\rm SC}\lpar k\comma \; j\rpar \vert } } \over L}\eqno\lpar 2\rpar $$

SC(*k*, *j*) is one element of the structural matrix and contains number of the fibres between regions *k* and *j*. In order to cancel out the effect of the ROI size, ${\rm SCG}{\rm M}_k$ is divided by *L* (volume of the ROI).

Eloquence score of a single fibre from the whole brain tractography is built upon GM areas that fibre intersects in brain volume: ${\rm ROIs} = \lcub {\rm G}{\rm M}_1\comma \; {\rm G}{\rm M}_2\comma \; \ldots \comma \; {\rm G}{\rm M}_m\rcub $ (*m* is the number of intersected areas). As such, functional score (${\rm FCW}{\rm M}_p$) and structural score (${\rm SCW}{\rm M}_p$) of fibre *p* are the mathematical average for the corresponding score of the associated regions (ROIs):

(3) $${\rm FCW}{\rm M}_p = \overline {{\rm FCG}{\rm M}_i} \quad {\rm where}\quad i \in {\rm ROIs}\eqno\lpar 3\rpar $$

(4) $${\rm SCW}{\rm M}_p = \overline {{\rm SCG}{\rm M}_i} \quad {\rm where}\quad i \in {\rm ROIs}\eqno\lpar 4\rpar $$

### 3.2 Path visualisation and tissue extraction

**Fig. 2:**
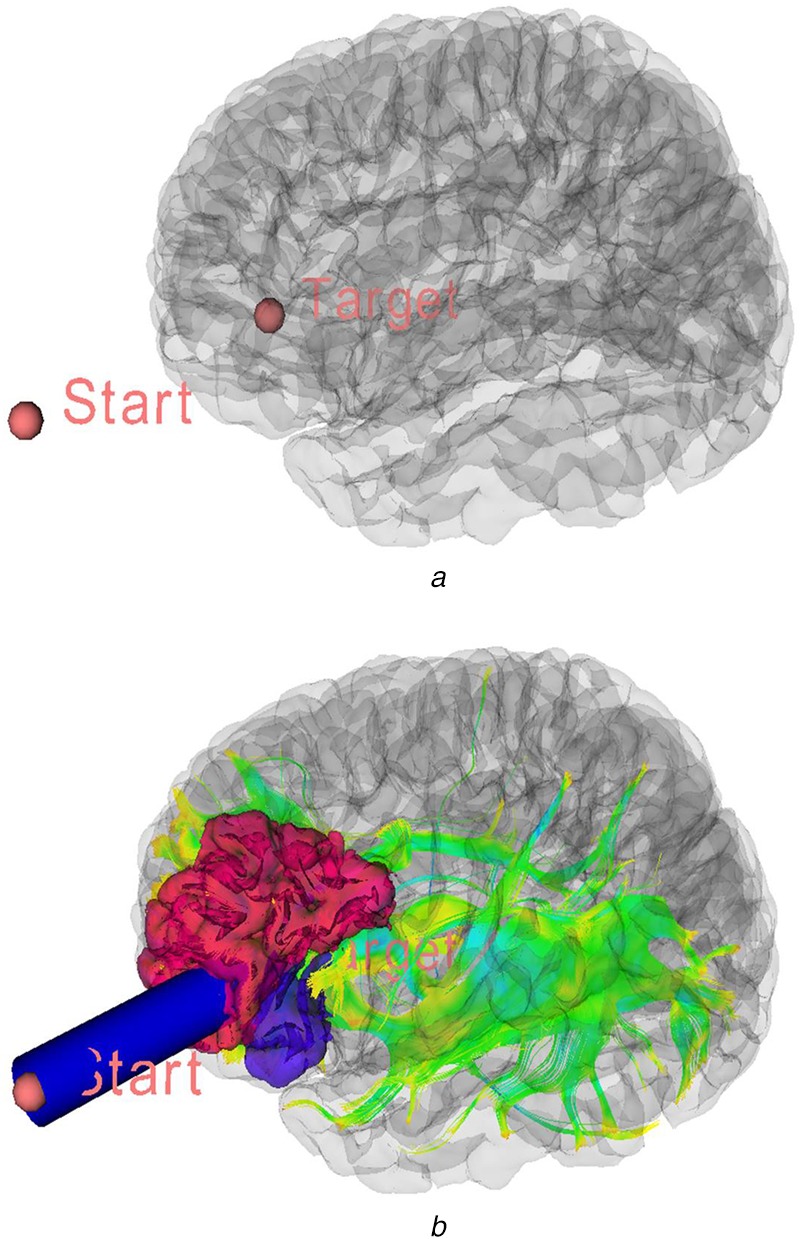
3D visualisation of a surgical path to a target lesion *a* Target landmark identified with a red sphere in 3D scene *b* Start point located outside of the skull depicted along with impacted WM and GM tissue

Surgical access path is modelled and visualised as a cylindrical tube (Fig. 2), which travels between two arbitrary points and its length and radius are adjustable. A sweeping spherical point cloud serves to scan through internal volume of cylinder to detect and store existing GM areas and WM fibres (Fig. 2) within the volume. At the same time, proposed algorithm (7) sums up eloquence scores of the relevant tissues. Main algorithm that has been used in this part is explained in previous works [18, 19].

### 3.3 Optimum path search

Solution space is formed by a 3D cube encapsulating the skull to capture all plausible paths towards any target point in the brain. Start points for the paths are located on five faces of the cube (all faces except the base, which overlaps with skull volume) with a grid of 1 cm spacing (a flattened version of search box is shown in Fig. 1(4)). For a single target point, search algorithm runs through all entry points on the box (Step (4)) and generates the path with GM and WM components in Step (7) and passes all the variables to Step (8). In which, total damage to the tissues is calculated by summing up the four components of eloquence score. In the next step, Step (9) reconstructs a volumetric box of the resulted scores and paints those points by numerical assessment of surgical outcome. In order to aggregate four components of the eloquence score, we decided to normalise them to a certain range by feature scaling (min–max scaling) followed by a summation of four components:

(5) $${X}^{\prime} = \displaystyle{{X - X_{\min }} \over {X_{\max } - X_{\min }}}\eqno\lpar 5\rpar $$

(6) $$\eqalign{T_{{\rm score}} ={\rm FCG}{{\rm {M}^{\prime}}}_{{\rm path}} + {\rm SCG}{{\rm {M}^{\prime}}}_{{\rm path}} + {\rm FCW}{{\rm {M}^{\prime}}}_{{\rm path}} + {\rm SCW}{{\rm {M}^{\prime}}}_{{\rm path}}} \eqno\lpar 6\rpar $$

**Fig. 3:**
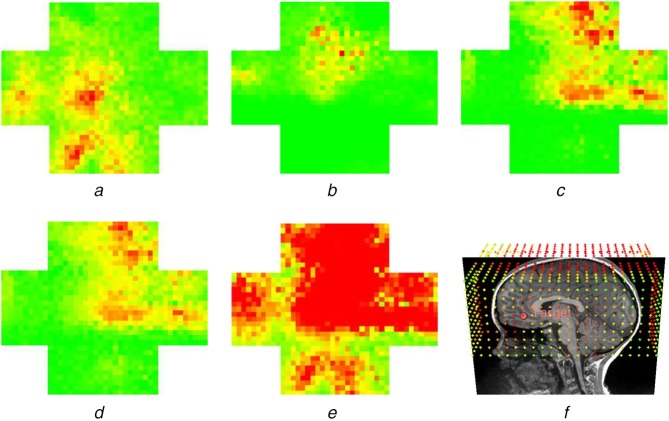
Visualisation of the optimal path search for a single target: Damages associated with *a* FCGM *b* SCGM *c* FCWM *b* SCWM *e* $T_{{\rm score}}$ *f* 3D reconstruction of $T_{{\rm score}}$ for image-guided interventions with target fiducial highlighted in red, green to red shows low to high damage

Equation (6) is equivalent to the total of GM volume and WM fibres normalised according to their statistical distribution and weighted with relevant eloquence score. To represent the outcomes of the proposed framework, a colour-coded risk map consisting of $T_{{\rm score}}$ for the candidate entry points is shown to the surgeon (Fig. 3*f*).

## 4 Experimental results

To analyse performance of the proposed method to the traditional approach of trajectory planning, seven targets were placed on various locations with medium depth and are shown in Fig. 1(3) by our clinical team. Then, for each target, one specialist neurosurgeon selected a trajectory based on 2D and 3D views relying on anatomical scan and cortical surface (generated by FreeSurfer [https://surfer.nmr.mgh.harvard.edu/]). We recorded initial response (indicated as *routine*). Following this step, neurosurgeon used this initial answer to search local minimums its vicinity and refine it to enhance the risk score ($T_{{\rm score}}$). Second step was performed under the guidance of 3D visualised scores resulted from the search. Radius of the tube representing the path was set to *r* = 5 mm for all of these trials. Results are represented in Table 1. Path length refers to the distance from the target to the cortical surface parallel to the assigned trajectory. Damage is the value obtained from (6) for the given path. *Damage score range* is the span of the values present in the search domain (3D box). Physical distance between projected points over the box is reported as *Euclidean distance* and angle between proposed and routine trajectory is reported as *Angular distance*. These experiments are carried out on an Ubuntu OS using a new 3D Slicer module which was developed for this study.

**Table 1:** Evaluation of the trajectories from proposed method to traditional decisions made by surgeons

Target number	Method	Path length, mm	Damage score range	Eloquence damage	Euclidean distance on the box, mm	Angular distance (deg)
1	routine	20	[0,560]	176	37	45
	proposed	22		58		
2	routine	15	[0,584]	240	38	65
	proposed	19		75		
3	routine	18	[0,641]	29	0	0
	proposed	18		29		
4	routine	21	[0,631]	108	69	49
	proposed	23		29		
5	routine	48	[0,570]	90	54	13
	proposed	36		37		
6	routine	49	[0,627]	194	26	15
	proposed	48		58		
7	routine	33	[0,585]	194	11	10
	proposed	28		70		
average	routine	29	[0,600]	147	34	28
	proposed	27		66		

## 5 Discussion

In the design of this study, the main assumption was that expert neurosurgeon can decide where approximately is the right entry point to the brain; given this assumption the goal was to provide a complimentary tool to fine-tune that decision by providing alternative access path in the vicinity of the initial decision that causes less harm to the brain networks. Table 1 provides the numerical results. Average of the path length for the proposed versus routine method are 27 and 29 mm. The main goal was to investigate the changes in damage, not the length. However, our results show that new method does not necessarily cause longer paths. Main objective of this study was to offer alternative trajectories in the proximity of the original trajectory whilst lowering estimated cognitive damage. This has been achieved by a small average physical difference (34 mm Euclidean and 28° angular) and improved cognitive damage with the new trajectory (66 compared to 148). In target number 3, initial response landed in a neighbourhood with minimal damage values and as a result, enhanced and routine trajectories are equal. Whereas, for target number 4, initial response was significantly altered to achieve a lower damage score, causing a significant distance between enhanced and routine approaches. Regarding the small to medium size brain tumours, since brain deformation is negligible, we predict that performance of the proposed method would be the same as healthy datasets tested in the current work. Moreover, we would like to clarify that the current experimental procedure is based on the fact that if an expert neurosurgeon agrees to change the initial decision, adjusted entry point is equally safe with the extra benefit of avoiding critical hubs of the brain networks.

## 6 Conclusion

The proposed eloquence metrics and estimated damage score are developed to quantitatively map graph theoretical network measures to their associated nodal areas. These anatomical hubs are perceived to play a central role in neural communications and information exchange across functional and structural networks [20]. Therefore, if resected, injured, or disconnected, may result in disruptions of network structure, leading to decline of cognitive performance or worse, debilitating motor, perceptual, or linguistic impairment. Accordingly, the surgeon can be supplied with anatomical counterparts of multimodal brain networks, without mentally being overloaded with connectome matrix data that does not provide explicit 3D structural information. Our current implementation utilises a typical network measure (unsigned weighted degree) [17]. It should be noted that the proposed eloquence scores are based on functional and structural connectivity, which have been shown in the last decade to correlate with actual cognitive abilities [21]. The main novelty of our approach is the visualisation and objective combination of both connectivity types. In the current format, this method is customised for planning an access path towards a medium-depth brain tumours or lesions; however, it can be modified for other neurological applications which depend on cognitive assessment of brain tissue. For deep-seated lesion or tumours, a simulation algorithm should accompany this method to take tissue deformations into considerations. Future work includes substitution and testing with other global or local network measures. We also consider to run comparative studies between entry points chosen by expert neurosurgeons and damage score maps generated by the proposed algorithm. Upon availability of clinical data, it would be worthwhile to carry out validation studies to evaluate accuracy of the score maps with regards to the post-operative outcomes.

## 7 Funding and declaration of interests

The work described in this paper was funded by the Natural Sciences and Engineering Research Council (NSERC) of Canada under grant A2600A04 (R. Eagleson) and the Canadian Institute of Health Research (CIHR) under grant MPI 325624 (S. de Ribaupierre). Hereby, we declare that the research was conducted in the absence of any commercial or financial relationships that could be construed as a potential conflict of interest.
